# Muscle Fiber Recruitment Characteristics in Trained Older Adults: An EMG Frequency Analysis During Voluntary Contraction

**DOI:** 10.3390/jfmk10030355

**Published:** 2025-09-17

**Authors:** Tatsuhiko Matsumoto, Yutaka Takamaru, Chikao Nakayama, Shuji Sawada, Shuichi Machida

**Affiliations:** 1 Murata Manufacturing Co., Ltd., 1-10-1 Higashikotari, Nagaokakyo-shi 617-8555, Kyoto, Japan; yutaka.takamaru@murata.com (Y.T.); chikao.nakayama@murata.com (C.N.); 2 Faculty of Health and Sports Science, Juntendo University, 1-1 Hirakagakuendai, Inzai 270-1695, Chiba, Japan; sh-sawada@juntendo.ac.jp; 3 Graduate School of Health and Sports Science, Juntendo University, 1-1 Hirakagakuendai, Inzai 270-1695, Chiba, Japan; 4 Institute of Health and Sports Science & Medicine, Juntendo University, 1-1 Hirakagakuendai, Inzai 270-1695, Chiba, Japan

**Keywords:** aging, surface electromyography, power spectral density, muscle fiber recruitment, resistance training, older adults

## Abstract

**Background:** Age-related declines in skeletal muscle quality and quantity contribute to frailty and sarcopenia, leading to physical dysfunction in older adults. In particular, the selective atrophy of fast-twitch fibers is closely linked to reduced lower limb strength and increased fall risk. This study aimed to evaluate the effects of habitual body weight resistance training on muscle fiber recruitment patterns during maximal voluntary contraction (MVC) and squatting movements using spectral characteristics of surface electromyography (EMG). **Methods:** Fifty healthy community-dwelling older adults (22 males, 28 females; aged 64–84 years) were categorized into exercise and non-exercise groups based on training habits. Surface EMG signals were recorded from the rectus femoris muscle during MVC, normal squat, half-squat, and squat movements. Power spectral density (PSD) was analyzed using Welch’s method and divided into frequency bands: type I (20–60 Hz), type IIa(1) (60–115 Hz), type IIa(2) (115–170 Hz), and type IIx (170–350 Hz). **Results:** While statistical significance was limited, the exercise group tended to show higher ratio PSDs in type IIa and IIx bands, and lower PSDs in the type I band during MVC. During half-squats, the non-exercise group exhibited higher contributions above 60 Hz, suggesting inefficient fast-twitch fiber recruitment. **Conclusions:** Habitual body weight resistance training may influence muscle fiber recruitment patterns in older adults. EMG spectral analysis offers a non-invasive means to detect age- and training-related neuromuscular adaptations.

## 1. Introduction

With the aging of the global population, maintaining muscle strength in older adults has become a major public health challenge. This age-related loss of muscle strength, referred to as “dynapenia”, is considered a component of sarcopenia and an independent clinical indicator of functional decline [1].

Lower limb muscle strength tends to decline more rapidly with age than upper limb strength, and is closely associated with mobility and independence. The thigh muscles, which are the largest muscles in the body and are essential for activities such as standing, walking, and stair climbing, are particularly vulnerable to age-related deterioration. Studies have shown a 50% reduction in thigh muscle fibers in older adults compared to young adults [2], accompanied by a preferential loss of fast-twitch fibers owing to impaired regenerative capacity [3]. These changes suggest that the loss of lower limb strength, especially in fast-twitch fibers, may serve as an early and critical indicator of functional decline in the aging population.

Reversibility is a key characteristic of age-related physical vulnerability, particularly frailty. Consequently, exercise interventions, especially resistance training, have received considerable attention as effective strategies for preventing the progression of dynapenia and sarcopenia and promoting recovery from frailty [4,5]. Recent meta-analyses have shown that resistance training significantly improves various functional outcomes in older adults, including grip strength, knee extensor strength, gait speed, and performance in the timed up-and-go test [6]. The effects of resistance training extend beyond increases in muscle strength and include alterations in muscle fiber composition and fiber-type-specific hypertrophic responses. Martel et al. (2006) conducted a high-intensity resistance training intervention under identical conditions in young and older adults and reported significant muscle strength gains in both groups [7]. They also observed that hypertrophy was more pronounced in fast-twitch fibers than in slow-twitch fibers. Similarly, Verdijk et al. (2009) implemented a 12-week high-intensity training program in older adults and observed an increase in the muscle fiber cross-sectional area and a significant increase in the number of satellite cells specifically in fast-twitch fibers [8].

Generally, the efficient recruitment of fast-twitch muscle fibers is believed to require high-intensity resistance training at ≥80% of the one-repetition maximum (1RM), with 8–12 repetitions per set, performed two to three times per week for at least 3 months [9]. However, in older adults, particularly those affected by sarcopenia or frailty, such high-intensity training may not always be feasible owing to cardiovascular or orthopedic risks including age-related impairments in muscle regenerative capacity. Therefore, in practical settings, there has been increasing interest in safer and more sustainable low-intensity, low-load resistance training, especially body weight-based exercises. Van Roie et al. (2013) reported that low-load training for muscular fatigue could elicit hypertrophic responses that are comparable to those induced by high-load training [10]. These findings suggest that sufficient muscular fatigue promotes the recruitment of a broad range of muscle fibers, including fast-twitch fibers, even at low exercise intensities. Moreover, body weight-based training does not require specialized equipment or facilities and can be continued at home, making it highly suitable for implementation in community- or home-based settings. Sawada et al. (2021) demonstrated an 11.1% increase in anterior thigh muscle thickness among older adults following a 12-week body weight resistance-training program, indicating that significant hypertrophic effects can be achieved without high-intensity intervention [11]. These findings indicate that skeletal muscle function, including the recruitment of fast-twitch fibers, can be improved in older adults under appropriate conditions.

Frequency spectrum analysis using electromyography (EMG) has attracted attention as a non-invasive method for estimating the types of muscle fibers recruited during muscle activity. In general, low-frequency components are more strongly associated with slow-twitch fibers, whereas high-frequency components are associated with fast-twitch fiber recruitment. Thus, the predominance or recruitment ratio of specific muscle fiber types can be estimated by observing the distribution and shifts in the spectral frequencies [12]. For example, in a study involving patients with chronic obstructive pulmonary disease, Casabona et al. reported that a spectral shift toward higher frequencies in the EMG signal could reflect the preferential recruitment of fast-twitch fibers or alterations in muscle fiber composition [13]. Furthermore, studies combining muscle biopsy and EMG analysis have shown a moderate correlation between the actual muscle fiber composition and the frequency and amplitude characteristics of EMG signals, thereby supporting the reliability of EMG spectral analysis in estimating muscle fiber recruitment patterns [14]. Changes in muscle fiber composition following resistance training have also been reported [15], indicating the potential applicability of EMG spectral analysis in evaluating the effects of training interventions.

Nonetheless, it remains unclear how such changes in muscle fiber composition are reflected in EMG spectral patterns during actual exercise and how the estimated types of recruited muscle fibers may differ before and after training. Studies involving older adults have provided limited quantitative evidence regarding the relationship between EMG spectral power in specific frequency bands and muscle fiber recruitment characteristics, and the findings have yet to be generalized. Moreover, scientifically established guidelines specifying the exercise intensity and frequency required to induce measurable changes in fiber recruitment patterns are lacking. Thus, while EMG spectral analysis is considered a promising non-invasive method for evaluating training-induced changes in muscle fiber recruitment, research validating its applicability and reliability in older populations remains scarce.

This study aimed to investigate the effects of habitual body weight resistance training on the power spectral density (PSD) characteristics of surface EMG signals from the rectus femoris muscle in older adults. We hypothesized that older individuals with regular training habits would exhibit a relatively higher PSD ratio in high-frequency bands associated with the recruitment of intermediate (type IIa) and fast-twitch (type IIx) muscle fibers than those without such habits do.

Through this analysis, we sought to determine whether continuous training in older adults is associated with the preferential recruitment of specific muscle fiber types, particularly fast-twitch fibers, during voluntary contractions. Furthermore, in cases where alterations in recruitment characteristics were observed, we aimed to explore their relationship with specific aspects of training habits and exercise intensity to establish practical indicators for promoting fast-twitch fiber recruitment. This study represents a novel attempt to evaluate the influence of training habits on muscle fiber recruitment patterns in older adults using EMG-based indices and is expected to provide new insights into the mechanisms underlying muscle function enhancement in the aging population.

## 2. Materials and Methods

### 2.1. Participants

The participants were healthy, community-dwelling, older Japanese adults residing in the cities of Inzai, Sakura, Shisui, Narita, and Tomisato in Chiba Prefecture, Japan. The exercise group (hereafter, “exercise group”) comprised individuals who had participated in community-based exercise programs and engaged in regular strength training over several years. Habitual training consisted of a structured body weight resistance program comprising nine exercises: squats, split squats, push-ups, heel raises, crunches, hip lifts, seated rows, shoulder presses, and arm curls. Sessions were typically performed 1–2 times per week. Each exercise was completed for 10–15 repetitions and 2–3 sets at an intensity corresponding to an RPE of 13–15, with gradual progression as tolerated. In contrast, the non-exercise group (hereafter, “non-exercise group”) was recruited through printed advertisements, such as flyers and posters, distributed or posted in local communities and public facilities. All participants completed a self-report questionnaire regarding their medical history and comorbidities. Individuals were excluded if they were unable to follow instructions, had chronic orthopedic conditions, or had health problems or diseases that moderately or severely impaired their walking ability. Ultimately, 50 healthy community-dwelling older adults (22 men, 28 women; age range, 64–84 years) were enrolled in this study. Written informed consent was obtained from all participants after receiving a detailed explanation of the study objectives and procedures. This study was approved by the Ethics Committee of Juntendo University (approval no. 2024-53).

### 2.2. Measurement Conditions

A questionnaire survey was administered to collect information on the participants’ sex, age, dominant leg, medical history, current exercise habits, and degree of sarcopenia progression. The questionnaire was distributed in either a paper- or web-based format, and self-administered responses were obtained. For body composition assessment, height was first measured, followed by body weight, skeletal muscle mass, and body fat mass using the InBody 440 device (InBody Co., Ltd., Seoul, Republic of Korea). Subjective fatigue was assessed using the Borg rating of perceived exertion (RPE) scale (6–20) before and after each exercise. The participants were asked to verbally report their perceived exertion immediately after the task in response to the examiner’s prompt. Muscle activity was recorded using surface EMG, targeting the rectus femoris as a representative muscle of the quadriceps femoris group. The EMG signals were acquired using an LP-iW2PAD system (Logical Products, Fukuoka, Japan). Bipolar surface electrodes (Ambu BlueSensor P-00-S, Ambu A/S, Ballerup, Denmark) with a center-to-center interelectrode distance of 20 mm were placed over the mid-belly of the rectus femoris muscle along the orientation of the muscle fibers. To ensure reproducibility, the electrode placement was standardized based on a written protocol. Before electrode attachment, the skin surface was carefully cleaned with an alcohol swab to remove excess oil and reduce impedance. The EMG sampling frequency was set at 1000 Hz. Handgrip strength was measured in the standing position with full elbow extension using a Smedley-type hand dynamometer (T.K.K.5401; Takei Kiki Kogyo, Niigata, Japan, now part of SANKA Co., Ltd., Niigata, Japan, as of 1 July 2024, operating as the Health Equipment Division). The participants were provided with standardized verbal encouragement during the measurements. Two trials were performed for each hand, alternating between hands to allow for rest between trials. The highest value among the four measurements was used for the analysis. Gait speed was used as an indicator of physical performance. The participants were instructed to walk at their usual pace along a 14 m corridor on a hard-surface floor, which consisted of a 2 m section for acceleration, a 10 m timed section for measurement, and a 2 m section for deceleration. Each participant completed two trials, and the faster of the two trials was used for analysis. Maximal voluntary isometric knee extension strength was assessed using a dynamometer (Takei Kiki Kogyo, Niigata, Japan) [16]. During the test, the participants were seated with their hip joints flexed at 90° (0° indicating full hip extension). The ankle was secured to the distal pad of the lever-arm. The knee joint angle was set to 90°, and the participants were instructed to perform a maximal isometric knee extension for approximately 5 s. A knee joint angle of 0° corresponded with a full knee extension. Before the maximal-effort trial, the participants performed several warm-up contractions (2–3 submaximal contractions and 1–2 near-maximal contractions). A single maximal isometric contraction was then performed, and the peak torque was analyzed. All procedures were performed according to a standardized protocol. After the strength test, the participants completed four exercise tasks under the supervision of experienced instructors, during which surface EMG signals were recorded.

- Task 1: Maximal voluntary contraction (MVC) of knee extension, as described above.
- Task 2: Half-squat. The participants stood with their feet shoulder-width apart and performed four repetitions of squats while maintaining the knee joint at approximately 120°. Each squat involved a 3 s descent and a 3 s ascent.
- Task 3: Normal squat. The participants performed four repetitions of squats to a depth in which the thighs were parallel to the floor, using the same 3 s descent and ascent rhythm as in the half-squat.
- Task 4: Normal squat hold. The participants held the squat position (thighs parallel to the floor) for 5 s while the EMG activity was recorded.

### 2.3. EMG Signal Processing

#### 2.3.1. Preprocessing

To evaluate the frequency characteristics of muscle activity during each exercise task, the recorded EMG signals underwent the following preprocessing steps: a 4th-order Butterworth bandpass filter with a passband of 10–499 Hz was used to remove motion artifacts and high-frequency noise. Subsequently, only time segments with stable muscle contractions were manually extracted from each EMG waveform. extraction was based on the criterion that the average amplitude should remain visually stable over time.

#### 2.3.2. Spectral Analysis

PSD analysis was conducted on the extracted stable segments of the EMG signals using Welch’s method. The resultant PSD was integrated across the entire frequency range and normalized to produce a relative power spectrum with a resolution of approximately 1 Hz.

The spectral indicator ratio PSD was calculated based on the PSD computed using Welch’s method. This measure, which represents the proportion of power within predefined frequency bands relative to the total PSD, was computed using the following formula:

(1) $$RatioPSD_{\left[f_{1},f_{2}\right]}=\frac{\sum_{f=f_{1}}^{f_{2}}PSD\left(f\right)}{\sum_{f=0.98}^{500}PSD\left(f\right)}$$

where

-
PSD(f) denotes the PSD at frequency *f*,
-
$f_{1}$ and $f_{2}$ represent the lower and upper bounds of the frequency band of interest (in Hz), respectively,
- and the denominator sums the power over the full frequency range from 0.98 Hz to 500 Hz.

This approach quantified the relative contribution of each frequency band to overall EMG activity.

There was ongoing debate regarding the optimal frequency band classification for estimating muscle fiber recruitment from EMG spectra. For instance, Wakeling and Rozitis (2004) demonstrated that the high-frequency components in the EMG spectrum of the quadriceps increased during ramp isometric contractions, as the force output progressively increased [17]. Focusing on the specific relationship between fiber types and frequency bands, a systematic review by Koenig et al. reported that in time-frequency analyses of lower-limb EMG during walking and running, slow-twitch fibers were primarily associated with low-frequency components (approximately 10–90 Hz), while fast-twitch fibers were linked to higher frequencies (60–270 Hz) [18]. Similarly, Hegedus et al. identified a fast-twitch fiber range of approximately 60–260 Hz [19]. In sustained submaximal contractions, Wakeling et al. observed distinct EMG spectral components in both the 40–60 Hz and 170–220 Hz bands [20]. Age-related differences in these characteristics have been previously reported. For example, Esposito et al. (1996) found that during isometric elbow flexion, older adults showed significantly lower mean EMG frequencies, with high-frequency peaks notably absent in younger individuals [21]. This suggests a reduced recruitment of large, fast-firing motor units (type IIx fibers) in elderly individuals. Based on these findings, Garcia et al. (2022) proposed that the following frequency ranges correspond to specific muscle fiber types: type I fibers, below 40–60 Hz; type IIa fibers, at 60–120 Hz; and type IIx fibers, at 170–220 Hz [22]. This frequency band classification is based on a multifaceted body of evidence, including single motor unit studies by Grimby et al. [23], EMG spectral analyses by Wakeling et al. [17], and electrical stimulation experiments conducted by Dreibati et al. [24].

In this study, the EMG spectrum was divided into the following frequency bands, each corresponding to a specific muscle fiber type:

- Type I fibers: 20–60 Hz.
- Type IIa(1) fibers (type IIa fibers with a profile closer to type I): 60–115 Hz.
- Type IIa(2) fibers (type IIa fibers with a profile closer to type IIx): 115–170 Hz.
- Type IIx fibers: 170–350 Hz.

Frequency components below 20 Hz were excluded from the analysis because of potential contamination by motion artifacts originating from electrode displacement or cable movement [25]. The upper bound of the type I range (60 Hz) and the lower bound of the type IIx range (170 Hz) were defined based on review by Garcia-Retortillo et al. [22], which showed that the activity of type I fibers primarily falls below 40–60 Hz, whereas that of type IIx fibers appears predominantly above 170 Hz. The upper limit of 350 Hz was selected based on the consideration that signal components above this range are more likely to be affected by environmental noise and fluctuations in the electrode impedance, thereby reducing the reliability of the muscle-derived signal interpretation [26,27]. A few previous studies have proposed a clearly defined boundary between type IIa and type IIx fibers, and the type IIa band was further subdivided into two parts (type IIa(1) and type IIa(2)) to allow for higher-resolution detection of potential shifts in recruitment patterns across the intermediate spectrum.

### 2.4. Statistical Analysis

Group comparisons were conducted to determine whether habitual training influenced muscle strength and EMG spectral characteristics. Participants who reported engaging in body weight resistance training at least twice per week were classified into the exercise group, whereas those who reported no resistance training were assigned to the non-exercise group. Participants who reported engaging in body weight resistance training once a week or less were excluded from the analysis (six males and four females). Additionally, participants with extremely high body fat were excluded to minimize potential errors in EMG signal acquisition. Individuals with body mass index (BMI) values rounded to 30 (to the nearest first decimal place) were excluded from the analysis (one male and one female).

For each frequency band, the ratio PSD was compared between the exercise and non-exercise groups using independent sample *t*-tests. Hedges’ g was calculated to evaluate the magnitude of the between-group differences. Hedges’ g represents the standardized mean difference between the two groups, adjusted for a small sample bias. This was computed by dividing the difference between the group means by the pooled standard deviation(SD) of the two groups, as follows:

(2) $$g=J\times \frac{{\bar{X}}_{1}-{\bar{X}}_{2}}{s_{\mathrm{pooled}}}$$

where $X_{1}$ and $X_{2}$ are the means of the two groups, $s_{\mathrm{pooled}}$ is the pooled SD, and *J* is the bias-correction factor. This adjustment reduced the upward bias in effect size estimates that could occur with small sample sizes, rendering Hedges’ g a more accurate alternative to Cohen’s d. The Interpretation of Hedges’ g followed conventional criteria; that is, values of 0.2, 0.5, and 0.8 were considered to indicate small, medium, and large effect sizes, respectively. All statistical tests were performed at a significance level of p<0.05 . Analyses were conducted using R software (version 4.2.2) [28].

## 3. Results

Table 1 presents the mean values and SDs of the physical and functional parameters stratified by sex and resistance training status.

Among the female participants, the exercise group demonstrated significantly greater maximal knee extension strength and body weight-adjusted strength than the non-exercise group ( p<0.05 ). In male participants, although these parameters did not reach statistical significance, the *p*-values were 0.13 and 0.11, respectively, indicating a trend toward significance. Furthermore, in males, significant differences or trends were observed between the groups in terms of height ( p<0.05 ) and BMI ( p<0.1 ). Overall, the exercise group demonstrated a tendency toward higher values across several physical and functional measures in both sexes.

**Figure 1:**
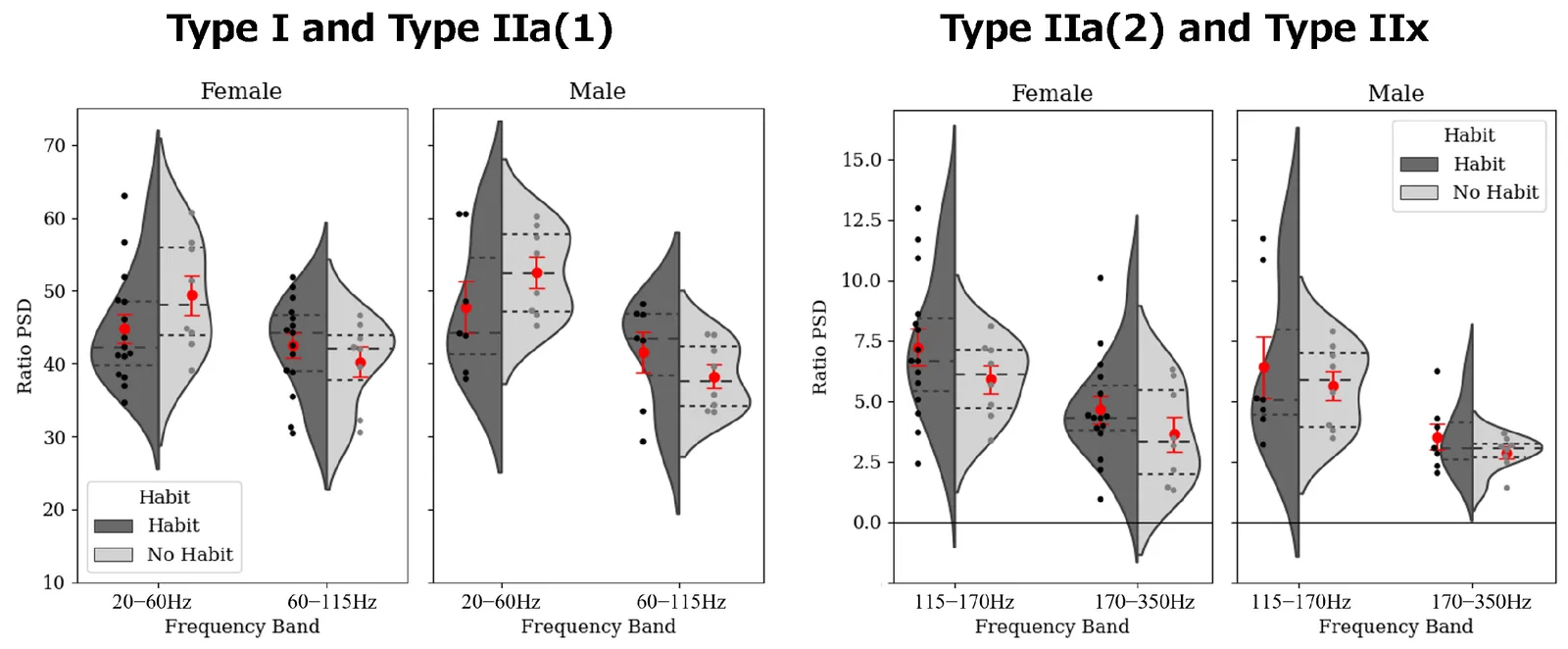
Ratio PSD of EMG frequency spectrum during MVC according to frequency band, resistance training habit, and sex. Violin plots display the distribution of ratio PSD recorded during MVC across four frequency bands associated with different muscle fiber types: type I, type IIa(1) (type IIa fibers closer to type I), type IIa(2) (type IIa fibers closer to type IIx), and type IIx. Plots are stratified according to resistance training habits (habit vs. no habit) and sex (male vs. female). Each violin plot illustrates the shape of the distribution, with overlaid individual data points (black dots) and group means ± standard error (red error bars). “Habit” indicates participants with regular resistance training; “no habit” indicates participants without such a habit.

Figure 1 illustrates the distribution of ratio PSD across different frequency bands during MVC, stratified by resistance training status and sex.

Visually, in the 20–60 Hz band, the non-exercise group exhibited higher mean values and a broader spread toward the upper range. Conversely, the exercise group showed higher mean values and greater upward dispersion in the 60–115 Hz band. These patterns were observed in both male and female participants. Independent *t*-tests were used to examine group differences in ratio PSD across each frequency band. No statistically significant differences were observed for any of the bands ( p>0.05 ).

**Figure 2:**
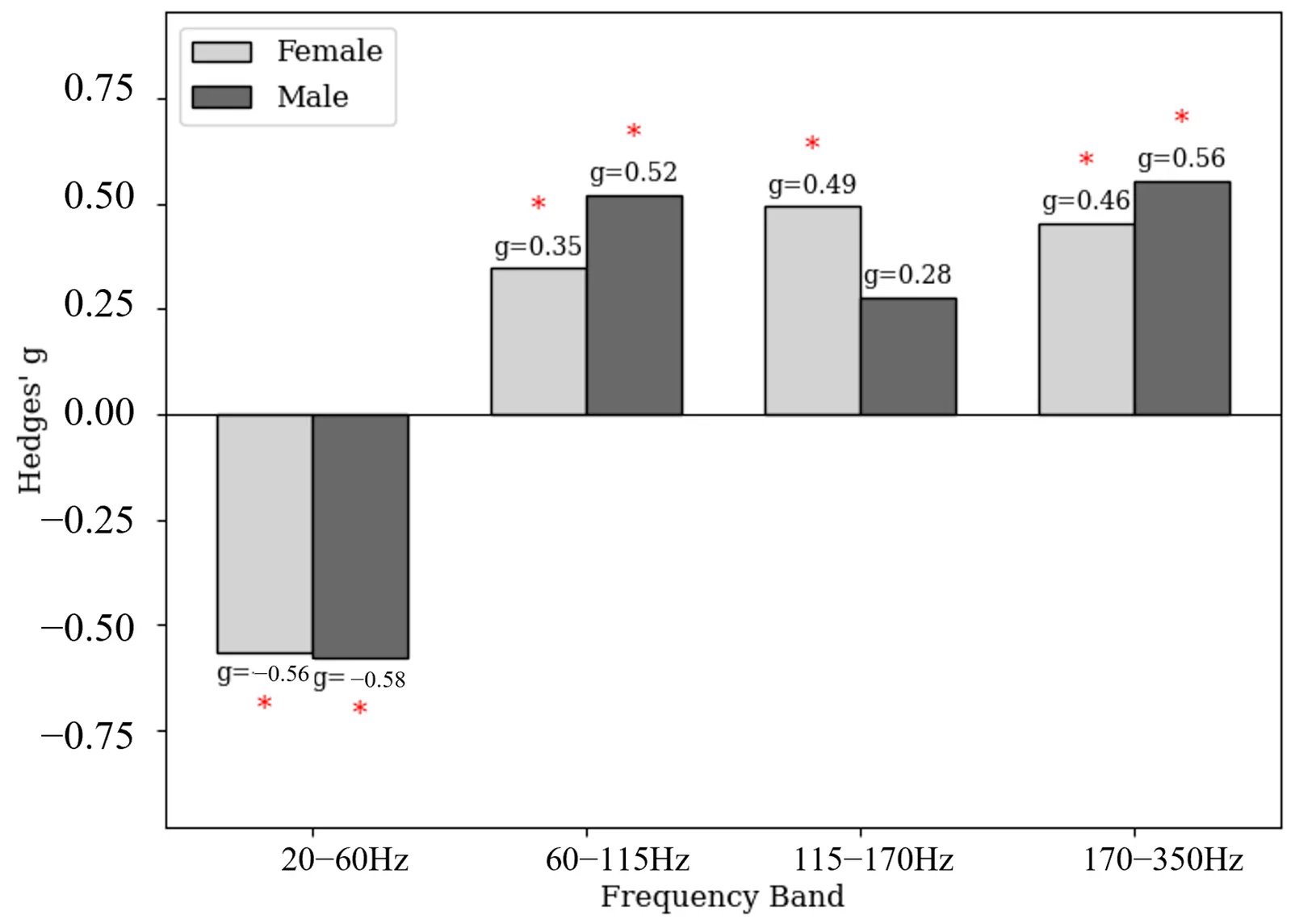
Hedges’ g effect sizes for between-group differences in EMG spectral power during MVC according to frequency bands and sex. Hedges’ g effect sizes are presented for the differences in normalized EMG spectral power (ratio PSD) between participants with and without a resistance training habit, calculated separately by sex and for each of four physiologically defined frequency bands: type I, type IIa(1) (type IIa fibers closer to type I), type IIa(2) (type IIa fibers closer to type IIx), and type IIx. Positive values indicate higher spectral power in the Habit group, whereas negative values indicate higher power in the No Habit group. An asterisk (*) indicates a medium or larger effect size ( |g|>0.3 ). “Habit” indicates participants with regular resistance training; “No Habit” indicates participants without regular resistance training.

Figure 2 displays the Hedges’ g effect sizes derived from the results shown in Figure 1, stratified by sex and frequency band.

Across all frequency bands, small-to-moderate or large effect sizes were observed. In the 20–60 Hz band, both males and females showed moderate-to-large negative effect sizes, suggesting a relatively higher ratio PSD in the non-exercise group. In females, large effect sizes were observed in the 115–170 Hz and 170–350 Hz bands. In males, moderate-to-large effect sizes were identified in the 60–115 Hz and 170–350 Hz bands. Notably, the direction of effect sizes was consistent between the sexes across all frequency bands, indicating a shared pattern of PSD distribution differences based on resistance training status.

**Figure 3:**
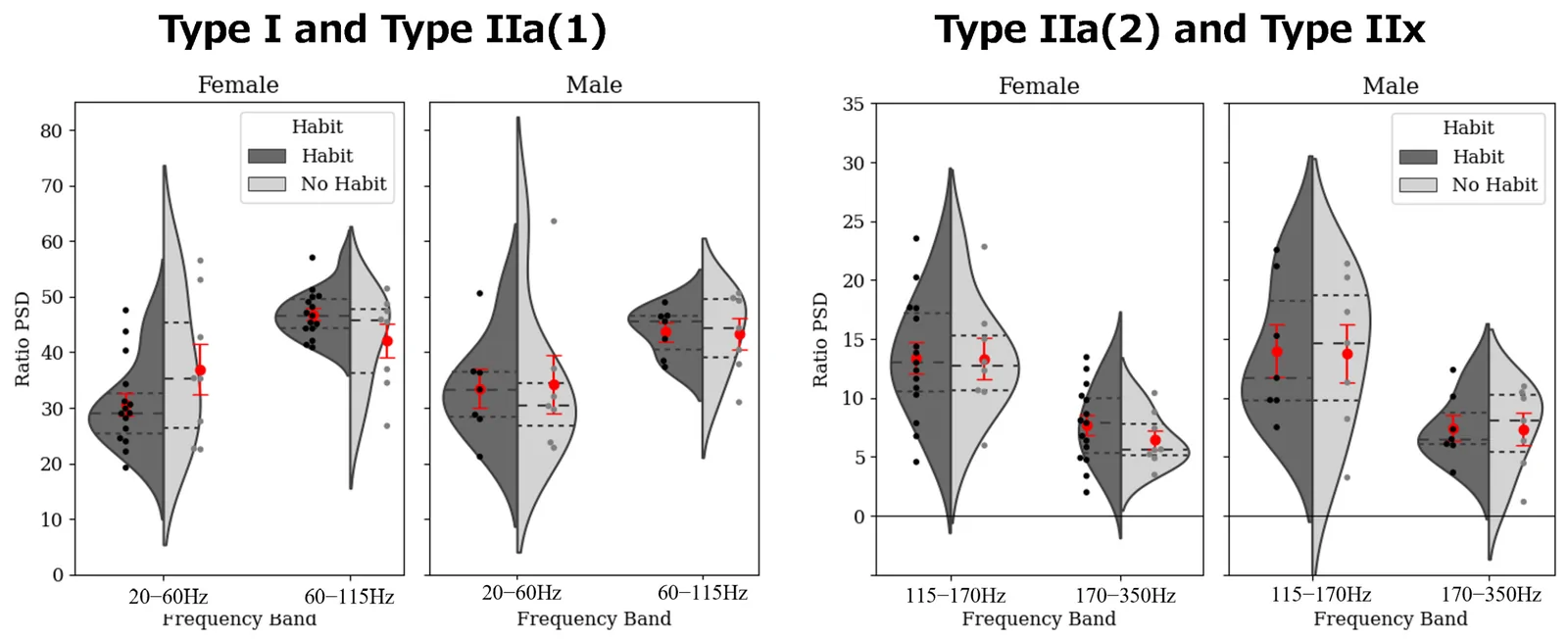
Ratio PSD of EMG frequency spectrum during isometric squat hold according to frequency band, resistance training habit, and sex. Violin plots display the distribution of ratio PSD recorded during an isometric squat hold across four frequency bands associated with different muscle fiber types: type I, type IIa(1) (type IIa fibers closer to type I), type IIa(2) (type IIa fibers closer to type IIx), and type IIx. Plots are stratified by resistance training habit (Habit vs. No Habit) and sex (male vs. female). Each violin plot illustrates the distribution shape, with overlaid individual data points (black dots) and group means ± standard errors (red error bars). “Habit” indicates participants with regular resistance training; “No Habit” indicates participants without such a habit.

**Figure 4:**
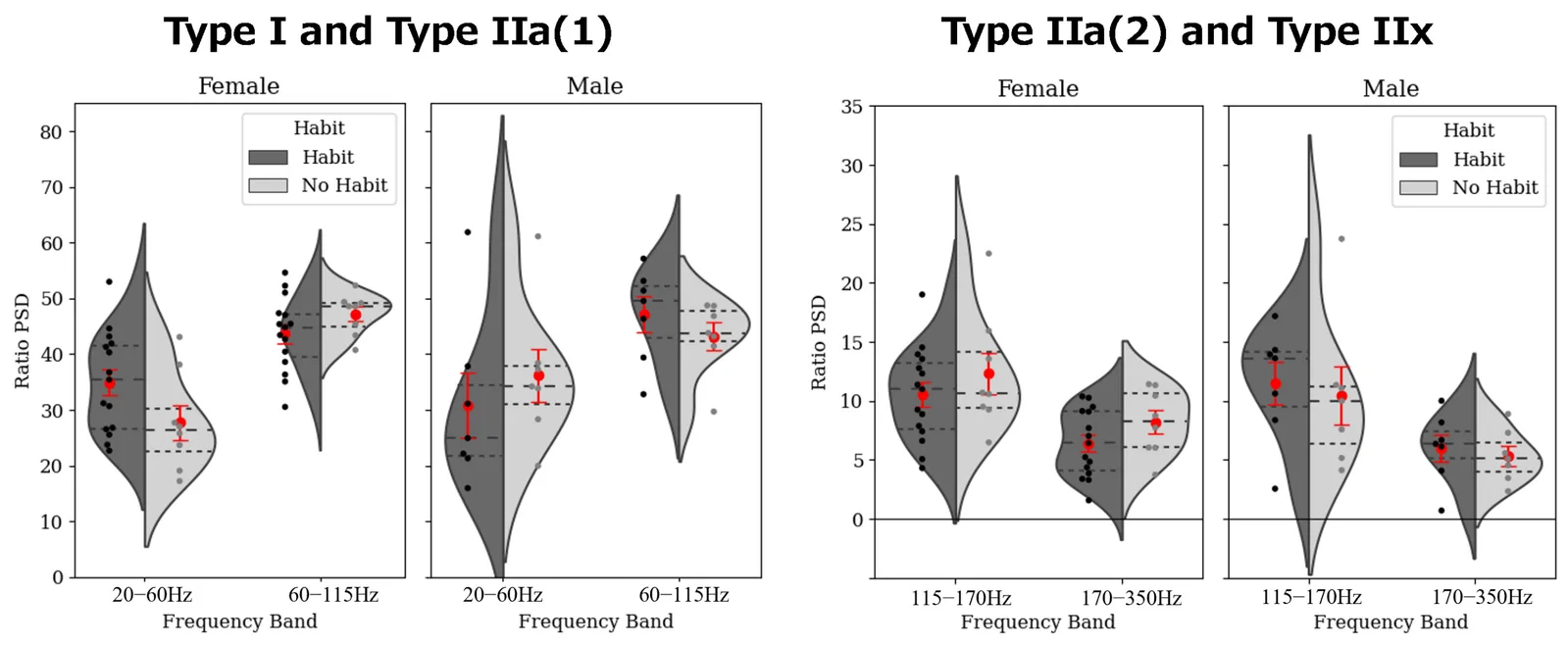
Ratio PSD of EMG frequency spectrum during a standard squat in the bottom position according to frequency band, resistance training habit, and sex. Violin plots display the distribution of ratio PSD recorded during a standard squat in the bottom position across four frequency bands associated with different muscle fiber types: type I, type IIa(1) (type IIa fibers closer to type I), type IIa(2) (type IIa fibers closer to type IIx), and type IIx. Plots are stratified by resistance training habit (Habit vs. No Habit) and sex (male vs. female). Each violin plot illustrates the distribution shape, with overlaid individual data points (black dots) and group means ± standard errors (red error bars). “Habit” indicates participants with regular resistance training; “No Habit” indicates participants without such a habit.

**Figure 5:**
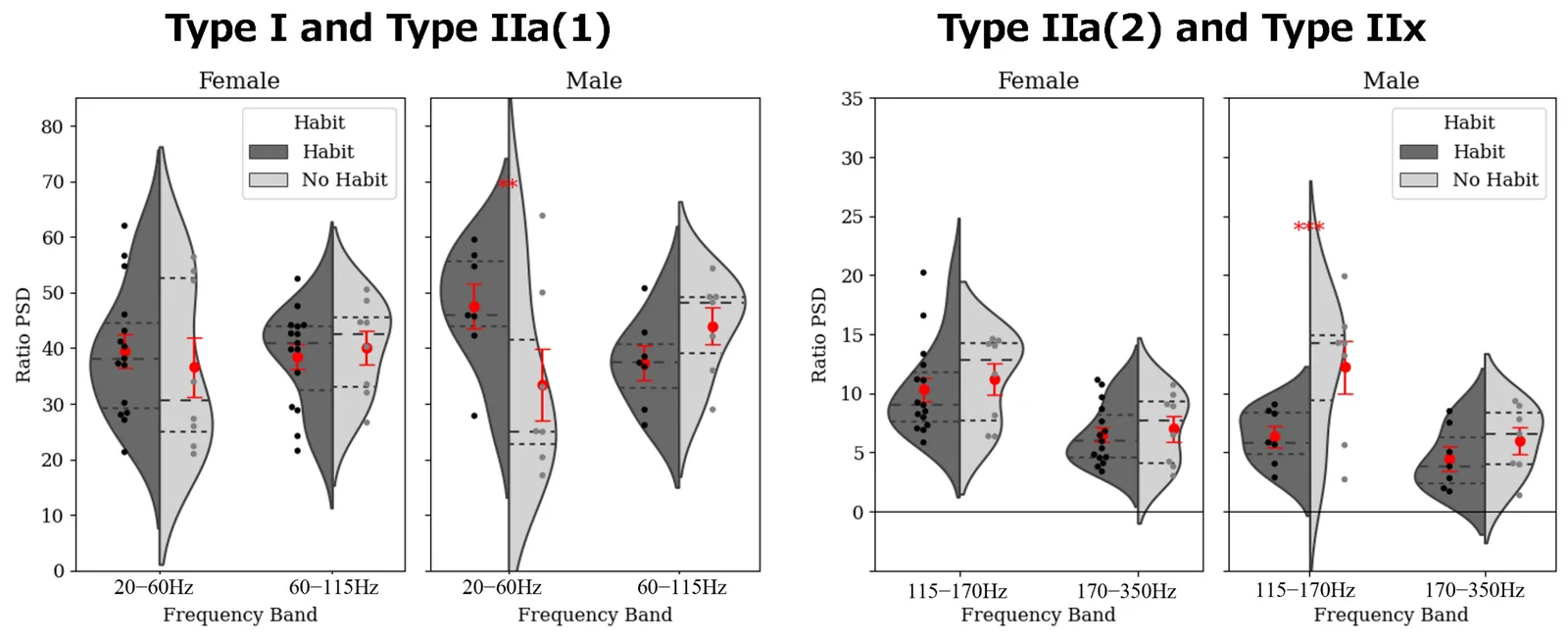
Ratio PSD of EMG frequency spectrum during partial squat in the bottom position according to frequency band, resistance training habit, and sex. Violin plots display the distribution of ratio PSD recorded during a partial squat in the bottom position across four frequency bands associated with different muscle fiber types: type I, type IIa(1) (type IIa fibers closer to type I), type IIa(2) (type IIa fibers closer to type IIx), and type IIx. Plots are stratified by resistance training habit (Habit vs. No Habit) and sex (male vs. female). Each violin plot illustrates the distribution shape, with overlaid individual data points (black dots) and group means ± standard errors (red error bars). “Habit” indicates participants with regular resistance training; “No Habit” indicates participants without such a habit. Asterisks above each plot denote between-group differences within each frequency band, based on Welch’s *t*-tests. Specifically, p<0.05 is marked with *** to indicate statistical significance, and p<0.1 is marked with ** to indicate a statistical trend.

Figure 3, Figure 4 and Figure 5 show the distributions of ratio PSD across different frequency bands during the squat hold, standard squat, and half-squat tasks, respectively, stratified by resistance training status and sex.

During the squat-hold task (Figure 3), the exercise group exhibited higher mean values in the 60–115 Hz band, with a markedly low variance observed in both sexes. Additionally, a visual difference in the distribution between the exercise and non-exercise groups was noted among females in the 20–60 Hz band.

During the standard squat task (Figure 4), an apparent reversal in the distribution trends between the exercise and non-exercise groups was observed across all frequency bands for both sexes. Among males, the non-exercise group showed higher mean values in the 20–60 Hz band, whereas the exercise group tended to show higher values in the higher frequency bands. Conversely, among females, the exercise group exhibited higher values in the 20–60 Hz band, whereas the non-exercise group tended to have higher values in the higher frequency bands.

During the half-squat task (Figure 5), a trend toward a higher ratio PSD was observed in the non-exercise group at frequency bands above 60 Hz for both males and females. Furthermore, independent *t*-tests indicated statistically significant differences for males in the 20–60 Hz and 115–170 Hz bands ( p<0.05 ).

## 4. Discussion

This study aimed to elucidate effects of habitual body weight-based resistance training on muscle fiber recruitment patterns in older adults by analyzing the PSD of surface EMG signals. PSD ratios across the defined frequency bands were computed from EMG signals obtained during various motor tasks in a cohort of 50 community-dwelling older adults. These ratios were compared between individuals with and without resistance training habits with further stratification by sex. Although statistically significant differences remain limited, the effect sizes and distributional trends suggested that the training group exhibited higher contributions in the high-frequency bands associated with types IIa and IIx, whereas the non-training group showed greater relative power in the low frequency band corresponding to type I. Although no significant between-group differences in the mean spectral values or distribution shapes were observed during MVC (Figure 1 and Figure 2), the trends in effect sizes and visual patterns implied distinct recruitment strategies between the groups. Specifically, the higher relative power in the 20–60 Hz band in the non-training group and in the 60–115 Hz band in the training group suggests a shift toward fast-twitch fiber recruitment in trained individuals. Notably, the directionality of these effect sizes was consistent across sexes, indicating a potentially universal influence of training on recruitment patterns, regardless of sex. Moreover, the relatively large effect sizes observed in females within the type IIa(2) and type IIx frequency bands may offer insights into sex-specific adaptations in fiber recruitment. Wakeling et al. previously reported spectral changes in the 40–60 Hz and 170–220 Hz bands associated with muscle fatigue, which aligned with the frequency ranges observed in our study despite our focus on non-fatiguing dynamic tasks [20]. Similarly, Garcia-Retortillo et al. (2020) demonstrated a marked reduction in PSD above 50 Hz following squat exercises in older adults, corroborating our findings that high-frequency power components are sensitive to muscle conditions in aging populations [29]. Importantly, this study extended these observations by demonstrating that habitual training alone, independent of acute fatigue, yielded measurable differences in high-frequency PSD components. These findings have implications regarding the development of more precise diagnostic markers for sarcopenia in clinical and research settings.

The finding that both males and females in the training group exhibited higher mean values and markedly reduced variance in the 60–115 Hz band during the squat-hold condition (Figure 3) suggests that resistance training may promote the development of a stable muscle recruitment pattern, particularly involving type IIa fibers. The consistent magnitude and dispersion across sexes further imply that long-term resistance training can effectively enhance neuromuscular control associated with type IIa fiber recruitment. During the standard squat task, a clear divergence emerged between the sexes; male participants showed a shift toward high-frequency components, whereas female participants exhibited a tendency toward lower-frequency dominance. Specifically, males demonstrated relatively reduced power in the 20–60 Hz band and increased power in the 60–350 Hz range. This pattern is consistent with the neural adaptations induced by resistance training, which facilitate the recruitment of high-threshold motor units (primarily type II fibers). Supporting this interpretation, Shin et al. (2006) reported linear increases in both integrated EMG and median frequency following a 12-week resistance training program, suggesting the enhanced involvement of fast-twitch fibers [30]. Furthermore, Del Vecchio et al. demonstrated that 4 weeks of isometric strength training significantly reduced motor unit recruitment thresholds and increased firing rates, indicating an enhanced neural drive to fast-twitch fibers [31]. In our study, these findings align with the elevated high-frequency PSD contributions observed in the trained male participants. Conversely, female participants exhibited higher relative power in the 20–60 Hz band and lower relative power in the 60–350 Hz band. Notably, several females in the training group displayed low MVC torque values despite their training status. According to a meta-analysis by Nuzzo et al. (2024), women tend to have a higher proportion of type I fibers and smaller cross-sectional areas of type II fibers than men [32]. These sex-related differences in muscle fiber composition may contribute to variability in training responsiveness, particularly with respect to fast-twitch fiber recruitment. These results underscore the need for individualized resistance training prescriptions that consider sex-specific neuromuscular characteristics and differential adaptive responses in older adults.

During the half-squat task, a higher proportion of PSD at frequencies above 60 Hz was observed in the non-training group, indicating that older adults without a history of resistance training rely on movement strategies that necessitate the recruitment of fast-twitch fibers, even under low-load conditions. Additionally, statistically significant differences were observed in the 20–60 Hz and 115–170 Hz bands among male participants, quantitatively demonstrating that habitual resistance training is associated with distinct muscle fiber recruitment patterns during dynamic exercise. These findings are consistent with those reported by Kwon et al. (2012), who found that older adults performing low-load movements exhibited a significantly lower proportion of EMG power in the 30–60 Hz range than younger adults [33]. Moreover, a reduction in this frequency band is correlated with postural instability, suggesting impaired neuromuscular control. Altogether, these observations imply that differences in muscle recruitment profiles may reflect reduced efficiency and immature neuromuscular control strategies during movement in untrained older adults.

This study had several limitations. First, because we employed a conventional frequency band analysis with uniform band definitions across all participants, the results captured only broad, group-level patterns. More detailed analytical approaches could potentially uncover finer individual differences in the EMG spectral characteristics that remain hidden in the current framework of this study. Second, the analyses did not account for individual-level factors such as age, BMI, or other potential confounders. Therefore, the observed associations should be interpreted cautiously. Incorporating these variables in future studies may clarify the unique contribution of habitual resistance training to neuromuscular adaptation. Third, the cross-sectional design of the study prevents causal inferences. Furthermore, we did not directly quantify the training load during each session. Developing low-burden systems to objectively monitor training intensity in older adults is essential to enhance reproducibility and comparability across studies. Fourth, the relatively small sample size, particularly after stratification by sex and training habits, limited the statistical power and may have masked subtle differences. Fifth, the participants were exclusively older adults, which restricts the generalizability of the findings to younger or clinical populations in the future. Despite these limitations, this study provides valuable initial evidence for the relationship between habitual resistance training and EMG spectral patterns in older adults. Addressing these limitations in future studies will improve the interpretability and translational value of the results, ultimately guiding the development of practical monitoring tools to support healthy aging through exercise.

## 5. Conclusions

This study investigated the influence of habitual body weight-based resistance training on muscle fiber recruitment patterns in older adults by conducting a PSD analysis of surface EMG signals obtained from the rectus femoris. Our findings suggest that regular training in older individuals may be associated with enhanced recruitment of fast-twitch muscle fibers, even during voluntary tasks. This study represents a novel, non-invasive attempt to quantitatively assess the relationship between habitual training and muscle fiber activation in aging populations using EMG spectral analysis. Future research should explore the quantitative associations between training frequency/intensity and recruitment patterns, as well as the longitudinal changes in neuromuscular adaptations. These insights may contribute to the development of more effective and individualized resistance training interventions for older adults.

## Figures and Tables

**Table 1:** Participant Characteristics According to Resistance Training Habit and Sex. This table shows the means and standard deviations (SDs) of key physical and functional variables according to sex and resistance training habits. Values are presented as mean ± SD. Group sizes (n) are indicated in the column headers. Asterisks indicate significance levels (** p<0.05 , * p<0.1 , trend only).

	Female (n = 23)			Male (n = 15)		
Variable	** RT≥2/Week (n = 16)**	**No RT (n = 8)**	** p -Value**	** RT≥2/Week (n = 8)**	**No RT (n = 8)**	** p -Value**
Age [years]	71.94 ± 3.49	72.0 ± 4.75	0.97	76.38 ± 3.54	76.62 ± 4.14	0.89
Height [cm]	152.33 ± 5.45	153.25 ± 6.59	0.71	160.79 ± 6.15	169.16 ± 5.68	0.01 **
Weight [kg]	55.11 ± 8.83	52.42 ± 4.42	0.42	65.84 ± 12.07	64.82 ± 8.28	0.84
BMI	23.7 ± 3.10	22.33 ± 1.46	0.15	25.31 ± 3.11	22.59 ± 1.81	0.05 *
Grip strength [kg]	23.81 ± 5.59	24.06 ± 3.67	0.94	36.25 ± 4.15	32.39 ± 4.87	0.22
Walking speed [ m/s ]	2.67 ± 0.3	2.75 ± 0.41	0.58	2.75 ± 0.4	3.02 ± 0.22	0.11
Maximal knee extension strength [ Nm ]	33.3 ± 9.43	26.83 ± 3.22	0.02 **	46.54 ± 13.64	37.77 ± 7.14	0.13
Maximal knee extension strength/body weight	0.61 ± 0.18	0.51 ± 0.05	0.04 **	0.7 ± 0.13	0.59 ± 0.12	0.11
Duration of resistance training [years]	5.5 ± 3.63	-	-	6.0 ± 2.48	-	-

RT: resistance training.

## Data Availability

Data supporting the findings of this study are available from the corresponding author upon reasonable request.
